# Heart rate variability analysis in sheep affected by transmissible spongiform encephalopathies

**DOI:** 10.1186/1756-0500-4-539

**Published:** 2011-12-14

**Authors:** Timm Konold, Gemma E Bone

**Affiliations:** 1 Pathology & Host Susceptibility, Neuropathology, Animal Health and Veterinary Laboratories Agency Weybridge, New Haw, Addlestone, Surrey KT15 3NB, UK

## Abstract

**Background:**

The function of the autonomic nervous system can be assessed by determining heart rate variability (HRV), which is impaired in some brainstem diseases in humans. Transmissible spongiform encephalopathies (TSEs) in sheep are diseases characterised by accumulation of disease-associated prion protein in the brainstem, including nuclei of the parasympathetic nervous system. This study was undertaken to assess whether analysis of HRV can be used as an aid in the diagnosis of TSEs in clinically affected, naturally or experimentally infected sheep.

**Findings:**

When HRV indices were compared between 41 clinical TSE cases (18 sheep infected with scrapie and 23 sheep infected with bovine spongiform encephalopathy), 11 control sheep and six sheep reported as scrapie suspects or dosed with BSE brain homogenate, which were not confirmed as TSE cases by postmortem tests, no significant differences were found between the groups. Median heart rate was significantly different but only when sheep were grouped by gender: it was higher in female TSE cases than in control sheep and higher in female than castrated male ovine classical BSE cases.

**Conclusions:**

HRV analysis was not useful as a diagnostic aid for TSEs of sheep.

## Background

Transmissible spongiform encephalopathies (TSEs) in sheep, such as scrapie or experimental bovine spongiform encephalopathy (BSE), are characterised by the accumulation of disease-associated prion protein (PrP^sc^) in the brainstem, particularly in the parasympathetic nucleus of the vagus nerve [1,2], which is used for the confirmatory immunohistochemical diagnosis of these prion diseases. The axons of neurons from this nucleus contribute to the formation of the motor component of the vagus nerve [3], which - together with the nucleus ambiguus in the brainstem - provide parasympathetic innervation of the cardiac sinoatrial node [4]. The function of the parasympathetic and sympathetic nervous system can be assessed by measuring heart rate variability (HRV), either by calculation of indices using statistical methods on R-R intervals (time domain analysis) or by spectral analysis of an array of R-R intervals (frequency domain analysis) derived from an electrocardiogram (ECG) [5]. Brainstem lesions have been shown to alter HRV in humans [6,7]. If PrP^sc ^accumulation in brainstem nuclei had any effect on their function one would expect detectable changes in HRV. We have previously been unsuccessful in using this method to aid the diagnosis of TSEs in cattle [8] but results in a pilot study in sheep suggested that HRV assessment may be useful as pre-clinical test for TSE infection [9]. As a result of the pilot study we evaluated whether clinically affected TSE-positive sheep could be distinguished from TSE-negative sheep by HRV analysis. Some of the data used had been presented at a conference at the European Society of Veterinary Neurology [10].

### Animals and disease confirmation

All procedures involving animals were carried out in accordance with the Animal (Scientific Procedures) Act 1986, under licence from the United Kingdom Government Home Office, which was granted following an internal ethical review process within the Veterinary Laboratories Agency (VLA).

The TSE status of each sheep was confirmed by postmortem tests, which included immunohistochemical examination (IHC) with monoclonal antibodies (mAbs) R145 (for all sheep except for eight sheep of New Zealand origin), 6H4 or P4 (for ovine BSE cases, to distinguish them from scrapie) according to established methods [11] and - for those sheep that were negative by pathological examination and the eight New Zealand-derived sheep -discriminatory Western immunoblot (Hybrid technique [12]).

Animals included in the study comprised 11 scrapie-free sheep, 18 sheep clinically affected with scrapie, one of which was experimentally infected following intracerebral inoculation with atypical scrapie brain, 23 clinically affected sheep with BSE, one of which was intracerebrally inoculated with L-type BSE brain, and six sheep that were inoculated with classical BSE brain or reported scrapie suspects unconfirmed by postmortem tests. Details of the sheep are given in Table 1. Although there was an overlap in the age ranges of the monitored sheep, the median age of BSE-affected sheep (Table 1) was significantly lower compared to the other groups (*P *< 0.0001, Kruskal-Wallis one-way ANOVA with subsequent Dunn's multiple comparison test, GraphPad Prism version 5, GraphPad Software, La Jolla, USA).

**Table 1:** Details of sheep with recorded electrocardiograms

Group	Number of animals	Gender: female, castrated male	Age range in months: min-max (median)	Breeds
Scrapie-free controls^1^	11	11, 0	33-58 (43.5)	Cheviot (5), Romney (3), Suffolk (3)

Scrapie^2^	18	17, 1	18-73 (51)	Romney (8), Suffolk (5), Charollais (1), Cheviot (1), Mule (1), Welsh Mountain (1),

BSE^3^	23	14, 9	21-54 (33)	Romney (11), Suffolk (8), Poll Dorset (3), Cheviot (1)

Inoculated with BSE/reported scrapie suspects, not confirmed^4^	6	5, 1	41-73 (46)	Romney (4), Suffolk (1), Welsh Mountain (1),

^1 ^Derived from a classical scrapie-free flock of New Zealand origin (N = 8 [13]) or from a UK classical scrapie-free Romney sheep flock (N = 3 [1]).

^2 ^Scrapie suspects reported by a farmer, inspected by a veterinary officer and transported live to VLA (N = 16), scrapie suspect from a research flock with high incidence of scrapie (N = 1 [14]) and a sheep intracerebrally inoculated with atypical scrapie (N = 1 [15]).

^3 ^Sheep orally inoculated with classical BSE brain homogenate (N = 21) or naturally infected in a BSE research flock (N = 1), some of which were included in a previous study [16], and one sheep intracerebrally inoculated with L-type BSE brain (kindly provided by Dr Cristina Casalone IZS Turin, Italy).

^4 ^Scrapie suspects reported by a farmer, inspected by a veterinary officer and transported live to VLA without disease confirmation (N = 3) and sheep orally inoculated with 0.5 g of BSE brain homogenate passaged through sheep (N = 3 [11]).

### Heart rate monitoring

Heart rate monitoring was carried out as described recently for cattle [8] with disposable skin-adhesive electrodes (Unilect, Unomedical Ltd. Stonehouse, UK) and added gel (Lectron II, Pharmaceutical Innovations, Newark, USA) to improve conductivity. The base-apex lead was chosen: the negative electrode was placed at the caudal angle of the left scapula, the positive electrode was placed at the intercostal space caudal to the left olecranon and the ground electrode was placed at the left paralumbar fossa. Recordings were made whilst the animal was restrained in a pen and lasted for at least 310 seconds. In the majority of cases, the ECG was recorded at the day of cull (49 sheep, 84%) or one day prior to cull (5 sheep, 9%); in two (3%) clinically affected sheep with classical BSE the recording was taken at 6 days and 21 days prior to cull respectively and in two sheep (3%) orally challenged with classical BSE brain but without disease confirmation the recording took place 1506 days prior to cull.

Recordings were amplified, digitised and processed using a micro 1401 MK II data acquisition unit and computer software Spike2 version 4 (CED, Cambridge, UK). Each R-peak of a QRS complex was marked. The HRV was determined from tachygrams of instantaneous heart rate, which were produced by plotting the length of the time between successive R peaks of a 5-minute ECG segment against cumulative time. Fast Fourier transform was performed on each tachygram, which separates the heart rate signal into its frequency components and quantifies them in terms of their relative intensity as power [17], using 1024 points to calculate the power spectrum. The range of the low frequency (LF) power band, representative of sympathetic and parasympathetic activity, was 0.04-0.15 Hz. The range for the high frequency (HF) power band, representative of parasympathetic activity at the respiratory frequency, was 0.15-1 Hz, which was based on a validation study of heart rate variability indices in sheep [18]. HF and LF power were expressed as absolute values and in normalised units (proportion of LF or HF power contributing to the total power minus the very low frequency power component below 0.04 Hz). In addition, the LF:HF power ratio was determined as a measure of sympathovagal balance [19]. Further variables determined were the mean heart rate (HR), the deviation of the mean R-R interval (in %) as a measure of sinus arrhythmia and the vasovagal tonus index (VVTI, natural logarithm of the variance in the RR-intervals [20] based on 300 R-R intervals).

### Comparison of data and discussion

**Figure 1:**
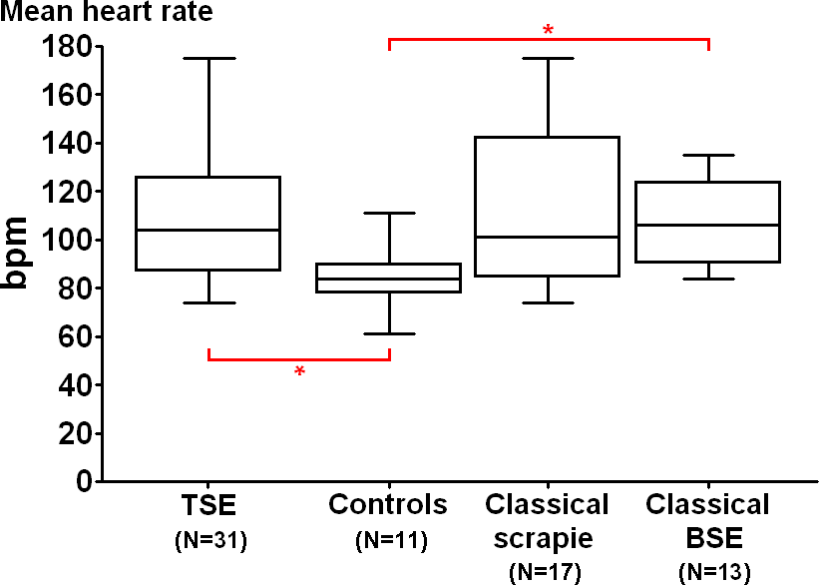
**Box plots of heart rates in female control and TSE-infected sheep further divided into classical BSE- and scrapie-infected sheep**. Box plots show the median, upper and lower quartiles and range. Significant differences (*P *= 0.02) are indicated by the red asterisk.

Table 2 shows the HRV indices for controls, TSE-positive sheep (clinical cases with confirmed scrapie or BSE) and suspects/dosed sheep that were not confirmed by postmortem tests. There were no significant differences for any of the indices between the groups (*P *> 0.05, Kruskal-Wallis one-way ANOVA, GraphPad Prism) and the ranges overlapped. This was in contrast to a previous study of HRV in scrapie-infected sheep based on accumulation of PrP^sc ^in recto-anal muscosa-associated lymphoid tissue, which described significant differences for the low frequency band between biopsy-positive and negative sheep even though the ranges between groups also overlapped [9]. The sheep in the reported study were not showing clinical signs, and the authors speculated that HRV analysis may be useful as a pre-clinical test for TSE infection. The TSE-infected sheep in the current study were clinically affected and it was expected that pathological changes in the brainstem, particularly PrP^sc ^accumulation, would be greater than in the pre-clinical phase and thus severe enough to cause changes in the function of autonomic nervous system that could be distinguished from healthy controls. In fact, the findings were similar to those made in BSE-affected cattle, which did not present with measurable changes in HRV compared to control cattle [8]. Significant changes in HRV indices of cattle were, however, associated with gender. To investigate whether this also applies to sheep we compared sheep groups of the same gender taking into account that the number of castrated male sheep was too small in some groups for any meaningful statistical analysis (see Table 1). When the HRV indices of all 31 TSE-positive female sheep were compared with the 11 female control sheep, significant differences (*P *= 0.009, Mann-Whitney *U *test, GraphPad Prism) were found for the median heart rate, which was higher (104 bpm, range: 74-175 bpm) in TSE-affected sheep than in controls (84 bpm, range: 61-111 bpm); see Figure 1. When separated by TSE type (classical scrapie versus classical BSE), BSE-affected female sheep had the highest median heart rate (106 bpm, range: 84-135; 13 sheep) compared to controls, which was significant (*P *= 0.02, Kruskal-Wallis one-way ANOVA with Dunn's multiple comparison test, GraphPad Prism), and scrapie-affected sheep (101 bpm, range: 74-175 bpm; 17 sheep); see Figure 1.

**Table 2:** Time and frequency domain indices in the three different groups

	Control sheep (N = 11)	TSE-positive sheep (N = 41)	TSE-negative suspects/dosed sheep (N = 6)
	**Median (range)**	**Median (range)**	**Median (range)**

LF power [ms^2^]	1704 (271.9-41841.0)	2200 (549.9-47466.0)	5568 (558.0-19038.0)

HF power [ms^2^]	367.8 (24.0-6240.0)	641.7 (117.1-14643.0)	936.6 (168.3-3928.0)

LF:HF power ratio	4.9 (1.7-11.3)	3.6 (0.7-40.7)	4.9 (2.5-9.3)

LF norm	83.1 (62.4-91.9)	78.2 (41.5-97.6)	83.0 (71.5-90.3)

HF norm	16.9 (8.1-37.7)	21.78 (2.4-58.5)	17.0 (9.7-28.5)

Mean heart rate [bpm]	84 (61-111)	101 (71-175)	103 (68-110)

VVTI	6.9 (4.4-11.3)	9.5 (3.5-19.8)	8.5 (7.0-11.3)

Deviation from mean interval [%]	42.8 (24.9-70.3)	45.9 (18.9-99.1)	63.2 (38.4-81.3)

**Figure 2:**
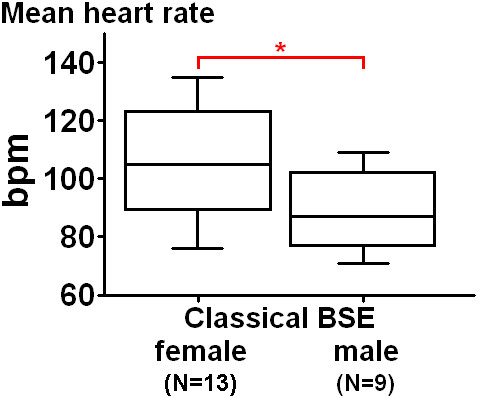
**Box plots of heart rates in female and male classical BSE-infected sheep**. Box plots show the median, upper and lower quartiles and range. The significant difference (*P *= 0.03) is indicated by the red asterisk.

Comparison between male (N = 10) and female (N = 13) classical BSE-affected sheep, which was the only group with sufficient numbers of sheep per gender, yielded again significant differences (*P *= 0.03, Mann-Whitney *U *test, GraphPad Prism) for median heart rate, which was higher in female sheep (106 bpm, range: 84-135 bpm) than in male sheep (89 bpm, range: 71-109 bpm), see Figure 2. The normal heart rate of adult sheep is 65-80 bpm although it may increase by more than 50% during handling before returning to near normal within 5-10 min [21]. Although sheep were only handled to attach the electrodes, ECGs were recorded whilst they were confined to a limited space to prevent unwanted animal movements and recording artifacts, which may still have caused a rise in the heart rate. As scrapie field cases were likely to be handled differently prior to transport to VLA we cannot exclude that the unfamiliar environment may have caused more stress and consequently a rise in the heart rate but there is no apparent explanation why the classical BSE-affected sheep, which previously had been familiar with human interactions during routine procedures, such as cleaning out pens, had the highest heart rates. We did not consider this to be the result of the comparatively younger age since all sheep in this study were adults. Scrapie-affected sheep have reportedly shown greater and more easily provoked rises of heart rate, up to 140-150 bpm [22], which is confirmed by the current study. However, this was not found to be the case in a clinical study of scrapie in Irish sheep, which presented with heart rates of 77 ± 20.2 bpm by auscultation, considered to be within the reference range [23]. The increased heart rate observed in TSE-affected sheep is contrary to the situation in cattle with BSE, which, despite agitation, present with a low heart rate suggestive of an increased parasympathetic tone [8,24]. If the higher heart rate in sheep was the result of an increased sympathetic tone or reduced parasympathetic tone as a result of a TSE, a median LF:HF power ratio or median LF norm higher than those in control sheep would have been expected, but this was not observed.

Our study included one sheep with atypical scrapie, which is characterised by far less PrP^sc ^accumulation in the brainstem compared to classical scrapie and, in the former, PrP^sc ^is predominantly found in the spinal tract nucleus of the trigeminal nerve [15,25]. However, all of the HRV indices obtained from this sheep were within the range established for the other (classical) scrapie cases.

**Figure 3:**
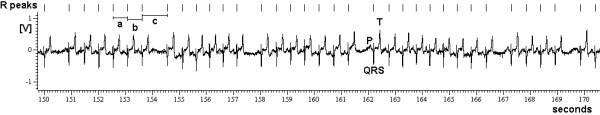
**Electrocardiogram of a classical BSE-infected sheep showing cardiac arrhythmia**. ECG of a 28 month-old Suffolk wether orally dosed with 5 g of ovine BSE brain homogenate (animal J196), which displayed clinical signs of BSE and presented with PrP^sc ^accumulation in the brain. P and T waves and the QRS complex are clearly identifiable. R-R intervals are marked above the ECG. The R-R interval c (0.94 sec) is almost twice as long as the previous two intervals, a (0.53 sec) and b (0.54 sec), which is close to the characteristics of sinus arrest.

In a separate study in Spain, ECG recordings of sheep with scrapie revealed cardiac arrhythmia at rest that was abolished by exercise [26]. The authors did not provide details of how arrhythmia was classified but when we compared the deviation of the mean R-R interval no significant differences were found between the groups although sheep with the highest deviation were found in the TSE-affected group (see Table 2 and Figure 3). Sinus arrhythmia, based on a variation of the R-R intervals of more than 10% [27], was observed in all sheep.

There is some variation in the band widths for low and high frequency power used by different researchers. Whilst the ranges in the current study were based on a previous study in lambs [18], a recent review suggested using a high frequency band of 0.20-0.40 Hz for sheep and goats, corresponding to a respiratory rate of 12-24 breaths per minute [28]. However, a study of breathing frequency in ruminants recorded a rate of 54 breaths per minute [29], equivalent to 0.9 Hz, which is within the reference range for adult sheep of 12-72 breaths per minute [30]. Others used a low frequency range of 0.032-0.138 Hz and 0.15-0.5 Hz for the high frequency band [9]. When we used similar ranges in the current study (limited by the equipment to 0.03-0.14 Hz for the low frequency band), the results did not differ (data not shown).

## Conclusions

HRV analysis was not able to distinguish TSE-affected sheep from healthy sheep or TSE-negative sheep (by postmortem tests) that were orally dosed with classical BSE brain homogenate or scrapie suspects. Separation by gender, however, revealed significant differences for the median heart rate, which was higher in female TSE-affected sheep than in female control sheep and - for sheep affected by classical BSE - higher in female than in castrated male sheep.

## Competing interests

The authors declare that they have no competing interests.

## Authors' contributions

TK and GEB recorded the electrocardiograms. TK carried out the analysis and drafted the manuscript. Both authors read and approved the final manuscript.
